# Effect of Pre-, Pro-, and Synbiotics on Biomarkers of Systemic Inflammation in Children: A Scoping Review

**DOI:** 10.3390/nu16030336

**Published:** 2024-01-23

**Authors:** Benjamin Momo Kadia, Stephen John Allen

**Affiliations:** Department of Clinical Sciences, Liverpool School of Tropical Medicine, Liverpool L3 5QA, UK; benjamin.kadia@lstmed.ac.uk

**Keywords:** prebiotic, probiotic, synbiotic, inflammation, children

## Abstract

Systemic inflammation plays a central role in many diseases and is, therefore, an important therapeutic target. In a scoping review, we assessed the evidence base for the anti-inflammatory effects of pre-, pro-, and synbiotics in children. Of the 1254 clinical trials published in English in Ovid Medline and Cochrane Library PubMed from January 2003 to September 2022, 29 were included in the review. In six studies of healthy children (n = 1552), one reported that fructo-oligosaccharides added to infant formula significantly reduced pro-inflammatory biomarkers, and one study of a single-strain probiotic reported both anti- and pro-inflammatory effects. No effects were seen in the remaining two single-strain studies, one multi-strain probiotic, and one synbiotic study. In 23 studies of children with diseases (n = 1550), prebiotics were tested in 3, single-strain in 16, multi-strain probiotics in 6, and synbiotics in 2 studies. Significantly reduced inflammatory biomarkers were reported in 7/10 studies of atopic/allergic conditions, 3/5 studies of autoimmune diseases, 1/2 studies of preterm infants, 1 study of overweight/obesity, 2/2 studies of severe illness, and 2/3 studies of other diseases. However, only one or two of several biomarkers were often improved; increased pro-inflammatory biomarkers occurred in five of these studies, and a probiotic increased inflammatory biomarkers in a study of newborns with congenital heart disease. The evidence base for the effects of pre-, pro-, and synbiotics on systemic inflammation in children is weak. Further research is needed to determine if anti-inflammatory effects depend on the specific pre-, pro-, and synbiotic preparations, health status, and biomarkers studied.

## 1. Introduction

Systemic inflammation plays a central role in the pathogenesis of several diseases. Deranged gut microbiota profiles, often referred to as “dysbiosis”, have been implicated in the onset and progression of some of these conditions, including gastrointestinal, metabolic, and neurological diseases [1,2]. This is linked to the crucial role of the gut microbiota in modulating local and systemic inflammatory and immune responses [2]. Consequently, modulation of the gut microbiota may contribute to the prevention or reduction of inflammation and, thereby, improved disease prevention and outcomes.

In this context, there has been a growing interest in the potential anti-inflammatory effects of pre-, pro-, and synbiotics. Prebiotic refers to a substrate that is selectively utilised by host microorganisms conferring a health benefit [3]. Probiotics are live microorganisms that, when administered in adequate amounts, confer health benefits on the host [4]. A synbiotic is a combination of live microorganisms and substrates selectively utilised by host microorganisms that confer a health benefit to the host [5]. Many different pre-, pro-, and synbiotics are available and in a variety of formulations [5,6].

The mechanisms by which pre-, pro-, and synbiotics may reduce inflammation are not fully understood, but it is suggested that they may modulate the gut microbiota, which can influence immune function and inflammation, and they may interact directly with immune cells and signalling pathways to reduce inflammation. Some probiotics produce metabolites, such as short-chain fatty acids, that have anti-inflammatory effects [7]. Furthermore, via colonisation resistance, some probiotics may impede the growth of pathogenic microbes and their products, thereby preventing or reducing inflammation [8].

Oral supplementation with pre-, pro-, and synbiotics has been studied in several diseases that have an inflammatory component, notably allergies, dermatitis, malnutrition, cancer, and gastrointestinal and respiratory diseases. A meta-analysis of 42 randomised controlled trials (RCTs) found that probiotic supplementation in adults with various diseases significantly reduced serum levels of pro-inflammatory biomarkers including high sensitivity C-reactive protein (hs-CRP), tumour necrosis factor-alpha (TNF- α), interleukin 6 (IL-6), interleukin 12 (IL-12), and interleukin 4 (IL-4), significantly increased serum levels of the anti-inflammatory cytokine interleukin 10 (IL-10), but did not affect concentrations of pro-inflammatory cytokines interleukin 1 beta (IL-1B), interleukin 8 (IL-8), interferon-gamma (IF-γ), and interleukin 17 (IL-17) [9]. A meta-analysis of 17 RCTs involving adults with diabetes found that probiotic supplementation significantly reduced the levels of pro-inflammatory markers CRP and TNF-α but not IL-6 [10]. A larger meta-analysis of 167 clinical trials involving adults and children found that pro- and synbiotics were effective in reducing CRP and TNF-α in both healthy and diseased subjects, although a disease-dependent reduction of other specific pro-inflammatory markers was also observed [11]. Contrary to these studies, a meta-analysis of seven RCTs found no significant effect of probiotics on CRP in trauma patients, but the small number of included studies with a combined sample size of 413 was insufficient to make firm conclusions [12]. It has been suggested that geographical origin, duration of consumption, and probiotic strain(s) that are consumed may influence the effect of probiotic-containing products on inflammation. A recent review of 14 RCTs found that probiotics were more effective in decreasing plasma concentrations of IL-6 and TNF-α and increasing IFN-γ in Asian male athletes using a single strain or when they were consumed for less than 4 weeks compared to adults from other geographical regions. However, the external validity of these findings is limited by the small sample size of 393 participants included in the review [13].

These systematic reviews have focused mainly on adult populations. The effects of pre-, pro-, and synbiotics in infants and children may differ from those in adults for several reasons. Perhaps most importantly, the gut microbiota is less complex, tends to have a different composition (being dominated by bifidobacteria) and develops over the first 2–3 years before the diverse microbiota characteristic of adults is established [14]. Also, adaptive and innate immune and inflammatory responses are developing in young children. Finally, interventions that reduce inflammation in early life may prevent cell and tissue damage and improve longer-term outcomes, especially for non-communicable diseases [15].

In our experience, the evidence from clinical trials that specific probiotics improve health is compromised by the large number of different probiotics that are evaluated, with few studies evaluating the same strains [16]. In addition, regarding trials assessing the effects of pre-, pro-, and synbiotics on systemic inflammation, we were uncertain as to the diversity regarding the participants recruited and the inflammatory markers measured. Therefore, rather than undertaking a systematic review, we sought to undertake a scoping review to assess the evidence base for pre-, pro-, and synbiotics in infants and children on reducing inflammation and identify research gaps [17].

Specific research questions were

1. How many RCTs have assessed the effects of pre-, pro-, and synbiotics on inflammation in infants and children?
2. What diseases have been studied?
3. What pre-, pro-, and synbiotic preparations have been evaluated?
4. What pro- and anti-inflammatory biomarkers have been evaluated?
5. Is there sufficient evidence to support meta-analysis?
6. What further research is required?

## 2. Materials and Methods

### 2.1. Search Strategy

Ovid Medline and Cochrane Library (PubMed) were searched for peer-reviewed articles published between 1 January 2003 and 30 September 2022. Articles retrieved from the search were saved on Mendeley desktop software (version 1.19.8), and their titles and abstracts were screened by B.M.K. Studies that fulfilled the eligibility criteria were retained for full-text review. Reference lists of eligible studies and previous reviews were hand-searched to identify additional eligible studies. Table 1 shows the search strategy used on Ovid Medline. The same search terms were used and tailored to the specific requirements of the Cochrane Library (PubMed).

### 2.2. Eligibility Criteria

Criteria based on population, intervention, comparator, outcome, and study design (PICOS) were used to determine the eligibility of studies for inclusion in the review. Based on these criteria, the following types of studies were included:

1. Population: infants and children aged 0–18 years
2. Intervention: prebiotics; probiotics (single- or multi-strain); synbiotics
3. Comparator: unexposed groups (routine treatment; control; no pre-, pro-, synbiotic)
4. Outcomes: blood/serum concentration of inflammatory/pro-inflammatory biomarkers or anti-inflammatory markers
5. Study design: RCTs
6. Reviews, case studies, conference abstracts, observational studies, and papers that were not in English were excluded.

### 2.3. Data Extraction and Analysis

A data extraction form was developed to standardise the data collection process. The data was extracted independently by B.M.K and S.A. The extracted data was saved in a Microsoft Excel 2016 spreadsheet and transferred to Microsoft Word. The data extracted included:

1. Publication details: first author, publication year, year of study, study design, study population, study location, sample size, characteristics of study participants (health status, age), study arms and interventions evaluated (pre-, pro-, and/or synbiotic) and main study findings.
2. Outcomes of interest: blood levels of pro-inflammatory or anti-inflammatory biomarkers.

Since the included studies evaluated the effect of pre-, pro-, and synbiotics in healthy subjects and/or subjects with specific diseases, the data were grouped by health status (healthy versus disease condition) and by intervention (pre-, pro-, and/or synbiotic) and synthesised descriptively. The initial categorisation of the data was performed by B.M.K. and then verified and refined by S.A. Meta-analysis of the effect of pre-, pro- and synbiotics on biomarkers of inflammation was not performed because of several sources of variability across the studies, notably variability in interventions (including differences in probiotic strains, prebiotic products, synbiotic products, and duration of intervention), and study populations (including differences in health status and disease conditions, control groups, and geographical location)

## 3. Results

**Figure 1:**
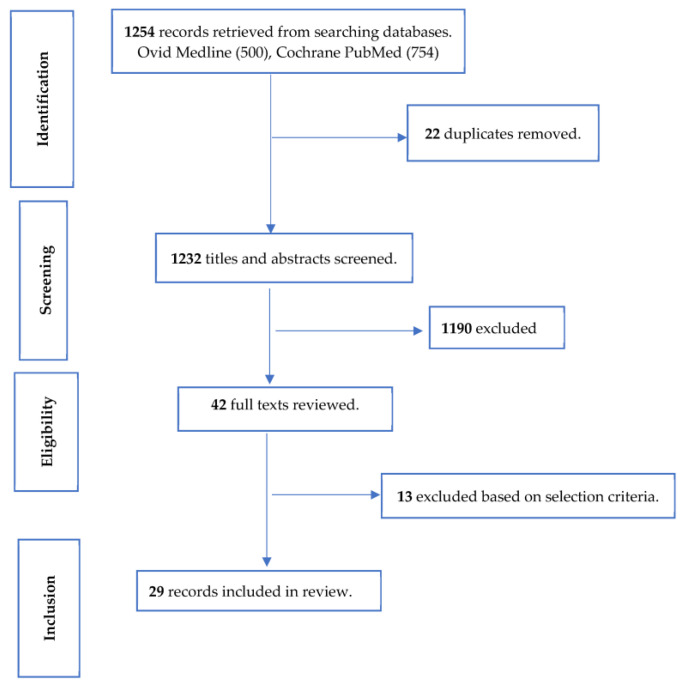
Selection of studies for review of the effect of pre-, pro-, and synbiotics on biomarkers of systemic inflammation in children.

Twenty-nine clinical trials with sample sizes ranging from 30 to 789 were retained from the search hit of 1254 articles. Figure 1 shows how the studies retained for the review were selected. These studies were conducted in many different countries and geographical regions, with 14 in Asia, 7 in Europe, 3 in North America, 3 in South America, 1 in Africa, and 1 in Oceania (https://ourworldindata.org/world-region-map-definitions; accessed on 1 December 2023).

### 3.1. Health Status and Pre-, Pro-, and Synbiotic Interventions

Six studies (n = 1552 children; range in sample sizes: 38–600) evaluated the effect of pre-, pro- and synbiotics on biomarkers of systemic inflammation in healthy children (Table 2). Three studies recruited neonates [18,19,20] and three older children [21,22,23]. One study evaluated a prebiotic [18], four a single probiotic [19,20,21,22], of which two studies evaluated *L. paracasei* subsp. *paracasei* F19 in the same daily dosage [20,22] and one synbiotic [19].

Twenty-three studies (n = 1154; sample sizes: 4–220) recruited children with various diseases, including atopic/allergic and autoimmune diseases, preterm infants, overweight/obesity, and severe illness (Table 3). Prebiotics were evaluated in three studies [24,25,26]. Two of these studies evaluated the Synergy 1 product (oligofructose-enriched inulin) [24,26] but in different doses and for different indications. Single-strain probiotics were evaluated in 16 studies [27,28,29,30,31,32,33,34,35,36,37,38,39,40,41,42], and multi-strain probiotics in 5 studies [31,36,43,44,45]. Nearly all probiotic studies evaluated lactobacilli and bifidobacteria, but few studies tested the same products. Three studies evaluated *L. plantarum*, but all used different strains [29,30,39]. Two studies evaluated *L. paracasei*, *L. fermentum*, or their combination, but for different indications [31,36]. Two studies assessed the multi-strain probiotic VSL#3 [44,45] but in different diseases. Only one study evaluated a synbiotic [46].

### 3.2. Biomarkers of Systemic Inflammation

A wide variety of pro- and anti-inflammatory cytokines and biomarkers were measured across the 29 trials. Pro-inflammatory biomarkers included chemokines, hs CRP, CRP, eosinophil sedimentation rate (ESR), granulocyte colony-stimulating factor (G-CSF), granulocyte-macrophage (GM)-CSF, interferon (IFN), IFN-α2, IFN-γ, IFN-γ–induced protein 10 (IP-10), IL-1α, IL-1β, IL-1 receptor antagonist (IL-1ra), IL-2, IL-5, IL-6, IL-7, IL-8, IL-12, IL-12p40, IL-12p70, IL-17, IL-17A, IL-31, lymphocyte subsets, monocyte chemoattractant protein (MCP)-1, macrophage inflammatory protein (MIP)-1, nerve growth factor (NGF), normal T cell expressed and secreted (RANTES), peripheral blood mononuclear cell (PBMC) count, and tumour necrosis factor (TNF)-α. Anti-inflammatory biomarkers included forkhead box P3 (Foxp3+) cells, IL-4, IL-10, IL-13, and TGF-β/TGF-β1.

The inflammatory biomarkers measured varied considerably between studies (Table 2 and Table 3). The most frequently measured pro-inflammatory cytokines were TNF-α (23 studies), IFN-γ (15 studies) and Il-6 (12 studies). The most frequently measured anti-inflammatory biomarkers were IL-10 (17 studies), IL-4 (10 studies) and TGF-β/TGF-β1 (9 studies).

Pro-inflammatory effects were evidenced by an increase in pro-inflammatory or a decrease in anti-inflammatory cytokines or biomarkers and vice versa for anti-inflammatory effects. The outcomes reported included differences in inflammatory biomarkers between study arms at the end of the intervention period, change from baseline in study arms and also differences in change from baseline in intervention versus control groups.

### 3.3. Effects of Pre-, Pro-, and Synbiotics on Biomarkers of Systemic Inflammation in Healthy Children

The effect of infant formulas containing galacto-oligosaccharides (GOS) plus two different concentrations of 2′-fucosyllactose compared with GOS alone from the ages of 5 days to 3 months in infants in the USA was assessed in a sub-study of an RCT [18]. At the age of 6 weeks, several pro-inflammatory cytokines were significantly reduced in the 2′-fucosyllactose groups compared with the GOS group, with greater effects in the lower dose group (0.2 versus 1.0 g 2′-fucosyllactose/L infant formula).

*L*. *paracasei* subsp. *paracasei* strain F19 administered from age 21 ± 7 days to 4 months in infants in China had both pro- and anti-inflammatory effects compared with standard formula [20].

The remaining studies, which tested a single-strain probiotic or synbiotics in neonates (one study) or a single-strain probiotic in older children (four studies), did not show any difference between study arms. However, inflammatory biomarkers were limited to either TNF-α and TGF-β1 [21] or CRP/high-sensitivity CRP [19,22,23] in these studies.

### 3.4. Effects of Pre-, Pro-, and Synbiotics on Biomarkers of Systemic Inflammation in Children with Diseases

Twenty-three studies reported the effect of pre-, pro-, and synbiotics on biomarkers of systemic inflammation in children with diseases (Table 3). Ten studies recruited children with atopic/allergic disorders. Of these, three reported anti-inflammatory effects [28,31,32], but in each study, this was limited to a single biomarker amongst those measured. Four studies reported both anti- and pro-inflammatory effects [29,30,34,35], and the remaining three studies found no significant difference between study arms [27,33,36]. The greatest number of studies were conducted in children with atopic dermatitis (five studies). Two of these studies evaluated *L. paracasei* [27,31], and two studies evaluated *L. rhamnosus* [28,32]; different preparations were used in each pair of studies, and one evaluated the probiotic heat-killed [27]. Different strains of *L. plantarum* were assessed in two studies [29,30], and only the measurement of IFN-γ and IL-4 was common in both studies. Two studies of children with allergic rhinitis [32,33] and two studies in asthma [35,36] evaluated different single- or multi-strain strain probiotics, limiting the evidence for efficacy. All nine studies of atopic/allergic disorders that assessed clinical outcomes reported positive effects in the intervention groups, including all three studies that reported both anti- and pro-inflammatory effects and all three studies that reported no effects on inflammation.

Five studies were conducted in autoimmune disorders [24,37,38,39,44]. In three studies that recruited children with coeliac disease [24,37,38], a single-strain probiotic reduced CD3^+^ and HLA-DR^+^ T lymphocyte counts and reported a beneficial clinical effect, but other inflammatory markers were not affected [37]. A multi-strain probiotic reduced TNF-α but did not report clinical outcomes [38]. A prebiotic did not reduce inflammatory biomarkers; clinical outcomes were not reported [24]. In the remaining two autoimmune studies, a single-strain probiotic did not affect inflammatory biomarkers in compensated or partially compensated nephrotic syndrome with dyslipidaemia [39], and a multi-strain probiotic resulted in both pro- and anti-inflammatory effects in enthesitis-related juvenile inflammatory arthritis [44]. Neither of these interventions improved clinical outcomes.

In two small studies in preterm infants, one study found that the anti-inflammatory effects of a single-strain probiotic were limited to an increase in TGF-β1 and other cytokines were not affected [40], while the other reported that a prebiotic did not affect CD4 and CD8 lymphocytes counts [25]. In a study of overweight/obese children in Canada, a prebiotic reduced IL-6, but other cytokines were not affected [26]. In two studies of children with severe illness, both conducted in intensive-care settings, results were more consistent, and both showed a clinical benefit. A single-strain probiotic in children with acute lung injury in China reduced both TNF-α and IL-6 [41], and a multi-strain probiotic in children with severe sepsis in India beneficially affected a number of inflammatory cytokines [45]. In single studies of other diseases, a multi-strain probiotic had beneficial effects on a number of inflammatory biomarkers and was also effective in preventing enterocolitis in children with Hirschsprung’s disease [43]. However, a synbiotic reduced only a single inflammatory marker in cystic fibrosis [46]. In a study of term infants with congenital heart disease in the USA, a single-strain probiotic increased IFN-γ without affecting other biomarkers [42]; this was the only study that reported an overall pro-inflammatory effect.

### 3.5. Effects on Inflammatory Biomarkers according to Intervention Tested

In four prebiotic studies, two reported an anti-inflammatory effect [18,26], and two showed no effects on inflammatory biomarkers [24,25]. In 20 studies evaluating a single-strain probiotic (including studies that included a single-strain probiotic arm), an anti-inflammatory effect was reported in 7 studies [28,31,32,37,38,40,41], both pro-and anti-inflammatory effects in 5 studies [20,29,30,34,35] and no difference between probiotic and control group in 7 studies [19,21,22,27,33,36,39] and 1 study reported a pro-inflammatory effect [42]. In the two studies of synbiotics, anti-inflammatory effects were reported in one study [46] and no difference in inflammatory biomarkers between groups in the other [19].

## 4. Discussion

The studies reported here were undertaken in markedly different populations and geographical regions (including richer and poorer countries) and on children with different health statuses varying from healthy children to a range of different diseases. Furthermore, many different pre-, pro-, and synbiotics were tested, and the selection of inflammatory biomarkers varied considerably between studies. Overall, the effects of these interventions on inflammatory biomarkers were mixed whether studies were grouped by health status or preparation tested. As a result of these multiple factors, the evidence base regarding the effects of these products on systemic inflammation is weak. Even though several studies have been conducted in atopic/allergic diseases, the variability in study designs limits the ability to pool data for meta-analysis.

There was minimal evidence that pre-, pro-, and synbiotics reduced inflammation in healthy infants and children. In the only study of a prebiotic in healthy children, 2′-fucosyllactose added to infant formula reduced several inflammation biomarkers in newborns in the USA. However, in studies of a single probiotic strain, equivocal effects were seen in a study of young infants [20] but not in another study of young infants [19] or studies in older children [21,22]. No effects on CRP were seen when a multi-strain probiotic [23] or different synbiotics [19] were administered to older children. It should be noted that in three of these studies, assessment of inflammation was limited to only either HsCRP or CRP, and this single index of acute inflammation may have missed effects on other biomarkers and longer-term effects. Given the early-life onset of systemic inflammation in non-communicable diseases [15], more studies of pre-, pro-, and synbiotics in healthy children assessing a broader range of inflammatory biomarkers are indicated.

A greater number of studies have been conducted on children with a range of diseases. Most studies have focused on children with atopic/allergic disorders. Although three studies reported anti-inflammatory effects [28,31,32], this was limited to only one of the measured biomarkers rather than a more general effect across multiple biomarkers. Interestingly, and in contrast to the biomarker findings, all nine studies that assessed clinical outcomes reported positive effects in the intervention groups, including studies that reported no significant differences in inflammation biomarkers between study arms. This suggests that beneficial modulation of the immune system relevant to atopic/allergic disorders was not captured well by the inflammatory biomarkers that were measured. In addition, whilst the clinical outcomes appear promising, the variability in pre-, pro-, and synbiotics evaluated in atopic/allergic disorders compromise the ability to undertake meta-analysis either for inflammation or clinical outcomes.

The effects of prebiotics and single- and multi-strain probiotics in autoimmune diseases were also mixed. As noted above, the evaluation of different products and variations in the inflammatory biomarkers measured compromise the pooling of data, including in the three studies of coeliac disease.

Similar constraints in evaluating the effects of pre-, pro-, and synbiotics were applied to the few studies conducted in other diseases. The possible exceptions were the two studies that evaluated either a single or multi-strain probiotic in severely unwell children and reported marked falls in several cytokines and improved clinical outcomes [41,45]. Further research on these preparations for severe illness is warranted.

Effects on inflammatory biomarkers were also mixed when studies were grouped by type of intervention tested. The additional effects of prebiotics beyond those of promoting the growth of “healthy” bacteria, such as direct modulation of host immune cells and antiadhesive actions against enteropathogens [47], may contribute to anti-inflammatory effects and merit further research. Regarding probiotics, the extent to which anti-inflammatory effects are shared across many organisms or are restricted to specific strains is unclear. Further studies with specific pre-, pro-, and synbiotic preparations are required to inform clinical practice.

Finally, the marked variability in inflammatory biomarkers measured in the studies included in this review, even those of similar conditions, and the discrepancy between laboratory and clinical findings in many studies suggest that more research is needed to determine optimal biomarkers of inflammation in specific diseases.

## 5. Conclusions

The evidence base for the anti-inflammatory effects of pre-, pro-, and synbiotics is weak and inconclusive. The evaluation of many different preparations in healthy children and across a broad range of diseases compromises meta-analysis. More studies are needed that evaluate the same pre-, pro-, or synbiotic preparations in the same populations and with harmonisation of inflammatory biomarkers to build the evidence base.

## Figures and Tables

**Table 1:** Search strategy for Ovid Medline.

Search #	Search Term(s) and Combinations
1	(probiotic * or prebiotic * or synbiotic *): ti, ab, kw
2	(infant * or neonat * or newborn * or p?ediatric or child *): ti, ab, kw
3	(‘Erythrocyte sedimentation rate’ or ESR or interleukin * or IL or ‘tumo?r necrosis factor’ or TNF or cytokine * or Orosomucoid or ORM or ‘alpha 1 acid glycoprotein’ or AGP or CRP or ‘C reaction protein’ or GlycA or ‘systemic inflammation’): ti, ab, kw
4	(Randomi?ed controlled trial): ti, ab, kw
5	(Review or protocol): ti, ab, kw
6	1 AND 2 AND 3 AND 4 NOT 5

* wildcard representing one or more characters in the search.

**Table 2:** Studies evaluating the effect of pre-, pro-, and synbiotics on systemic inflammation in healthy children.

Author, Year	Study Design and Population ^1^	Intervention(s)	Biomarker(s)	Significant Intervention Effects		
				Anti-Inflammatory	Pro-Inflammatory	Overall Effect ^2^
**Prebiotic studies**						
Goehring et al., 2016 USA [18]	Sub-study; 3-arm RCT; 113 term newborns	Prebiotic: Infant formulas containing 2.4 g total oligosaccharides/L (control: GOS only; experimental formulas: GOS + 0.2 or 1.0 g 2′-fucosyllactose/L [2′FL/L]) from 5 days to 4 months	IFN-α2; IFN-γ; IL-1α; IL-1β; IL-6; IFN-γ–induced protein 10 (IP-10); TNF-α; IL-1 receptor antagonist (IL-1ra); regulated upon activation, normal T cell expressed and secreted (RANTES); IL-10; lymphocyte count; PBMC phenotyping and stimulation ex vivo; measured at 6 weeks of age	Lower IL-1α, IL-1ra, IL-1β, IL-6 and TNF-α (*p* ≤ 0.05) in both 2′FL/L groups. Lower TNF-α, IFN-γ and IL-6 in 0.2 g 2′FL/L group in culture supernatants of RSV-stimulated PBMCs ex-vivo (*p* ≤ 0.05). Greater percentage of sub-G0/G1 CD56+ cells in 0.2 g 2′FL/L group (*p* ≤ 0.05).	-	↓
**Probiotic studies** **—** **single strain**						
Li et al., 2021 China [20]	3-arm RCT: 495 infants aged 21 ± 7 days randomised to formula with F19, milk fat globule membrane or standard formula	Probiotic: *L*. *paracasei* subsp. *paracasei* strain F19 10^8^ cfu/L from age 21 ± 7 days until 4 months	IL-2, IL-6, IL-17A, IFN-γ, TNF-α; TGF-β1, IL-4 measured at 4 months of age	Lower IFN-γ (*p* = 0.008)	Higher IL-2 (*p* = 0.024)	↑/↓
Kusumo et al., 2019 Indonesia [21]	4-arm RCT: 38 children aged 12–24 months randomised to placebo, probiotic, zinc, and probiotic + zinc	Probiotic: *L. plantarum* IS-10506 of ‘dadih’ origin 2.3 × 10^10^ cfu/g/d for 90 days	TNF-α and TGF-β1 pre-/post-treatment ratio measured at baseline and end of study	-	-	→
Karlsson et al., 2015 Sweden [22]	2-arm RCT: 120 children aged 4–13 months	Probiotic: *L. paracasei* subsp. *paracasei* F19 (LF19; deposition number LMG P-17806) 10^8^ cfu daily from age 4 to 13 months	HsCRP at 8–9 years of age	-	-	→
**Probiotic studies** **—** **multi-strain**						
Larnkjaer et al., 2021 Denmark [23]	2-arm RCT: 186 children expected to start daycare at age 8–14 months	Probiotic: *L. rhamnosus* LGG^®^ plus *B. animalis* subsp. *lactis*, BB-12^®^ each 10^9^ cfu daily from up to 12 weeks before starting daycare for 6 months	CRP measured at 6 months	-	-	→
**Synbiotic/probiotic studies**						
López-Velázquez et al., 2015 Mexico [19]	5-arm RCT: 600 infants aged 20 ± 7 days	Prebiotics: Infant formula with fructans obtained from *Agave tequilana* var Weber: Metlin^®^ and Metlos^®^. Probiotic: *L. rhamnosus* (LR) 0.3 × 10^7^ cfu. Group 1: Synbiotic (LR + Metlin^®^ + Metlos^®^); Group 2: Synbiotic (LR + Metlin^®^); Group 3: Synbiotic (LR + Metlos^®^); Group 4: Probiotic (LR; Group 5: infant formula only; daily to age 3 months	CRP measured at baseline and 3 months	-	-	→

^1^ Only results from randomised study participants included. ^2^ ↑ = pro-inflammatory effect; ↓ = anti-inflammatory effect; ↑/↓ = both pro- and anti-inflammatory effects; → = no effect observed. RCT—randomised controlled trial; PMBC—peripheral blood mononuclear cells; GOS—galacto-oligosaccharides.

**Table 3:** Studies evaluating the effect of pre-, pro-, and synbiotics on systemic inflammation in children with disease conditions.

Author, Year, Country	Study Design and Population	Intervention(s)	Biomarker(s)	Significant Intervention Effects			
				Anti-Inflammatory	Pro-Inflammatory	**Overall Effect ^1^**	**Clinical Effect ^2^**
**Atopic/allergic disorders: atopic dermatitis**							
D’Auria et al., 2021 Italy [27]	2-arm RCT: 58 children aged 6–36 months with atopic dermatitis	Probiotic: 8g rice-dried powder containing heat-killed *L. paracasei* CBA L74 powder daily for 12 weeks	G-CSF, GM-CSF, IFN-γ, IL-1β, IL-2, IL-5, IL-6, IL-7, IL-8, IL-12, IL-17A, MCP-1, MIP-1β, TNF-α, IL-4, IL-10, and IL-13 measured at 12 weeks	-	-	→	Yes
Jeong et al., 2020 Korea [28]	2-arm RCT: 66 children aged 1–12 years with atopic dermatitis	Probiotic: tyndallized *L. rhamnosus* (RHT3201) 10^10^ cfu daily for 12 weeks	Eosinophil cationic protein (ECP), CRP, IL-31, TNF-α, chemokines; IL-4, IL-10 measured at 6 and 12 weeks	Improved ECP level at week 12 (*p* = 0.022)	-	↓	Yes
Prakoeswa et al., 2017 Indonesia [29]	2-arm RCT: 22 children aged 0–14 years with mild and moderate atopic dermatitis	Probiotic: microencapsulated *L. plantarum* IS-10506 10^10^ cfu/day twice daily for 12 weeks	IFN-γ, IL-17; IL-4, forkhead box P3 (Foxp3+)/IL-10 ratio; CD4+ expression of IL-4, IFN-γ, Foxp3+/IL-10 ratio, and IL-17 measured at 12 weeks	IFN-γ decreased more in probiotic group (*p* = 0.006). CD4+ expression of IFN-γ fell in probiotic but not control group. Increased Foxp3+/IL-10 ratio in probiotic compared with control group (*p* = 0.001).	IL-4 decreased more in probiotic group (*p* < 0.001)	↑/↓	Yes
Han et al., 2012 Korea [30]	2-arm RCT: 79 children aged 12 months to 13 years with atopic dermatitis	Probiotic: *L. plantarum* CJLP133 0.5 × 10^10^ cfu twice daily for 12 weeks	TNF-α, IFN-γ, and IL-4 measured at 2 and 14 weeks	IFN-γ decreased from baseline (*p* < 0.001) and was lower in the probiotic than placebo group at 14 weeks (*p* < 0.05)	IL-4 decreased from baseline (*p* = 0.049)	↑/↓	Yes
Wang et al., 2015 Taiwan [31]	4-arm RCT: 220 children aged 1–18 years with moderate-to-severe atopic dermatitis	Probiotics: *L. paracasei* (LP; 2 × 10^9^ cfu) or *L. fermentum* (LF; 2 × 10^9^ cfu) or both (4 × 10^9^ cfu) daily for 3 months	IFN-γ, TNF-α, and TGF-β, IL-4 measured at baseline and 3 months	Increased IL-4 from baseline (*p* = 0.04)	-	↓	Yes
**Atopic/allergic disorders: allergic rhinitis**							
Jerzynska et al., 2016 Poland [32]	3-arm RCT: 100 children aged 5–12 years with allergic rhinitis and sensitivity to grass pollen receiving sublingual immunotherapy	Probiotic: *L. rhamnosus* GG (Dicoflor 30) 3 billion live cultures daily for 5 months	CD4^+^CD25^+^Foxp3^+^ (forkhead box P3) cells, Toll-like receptor (TLR) 4, IL-1, IL-6, TNF-α, TLR activation, IL-10, and TGF β-1 in cell supernatants of grass-stimulated PBMCs measured at baseline and 5 months	Increased forkhead box P3 cell induction at 12 months (*p* < 0.05)	-	↓	Yes
Lin et al., 2014 Taiwan [33]	2-arm RCT: 60 children aged 6–13 years with perennial allergic rhinitis receiving levocetirizine (antihistamine)	Probiotic: *L. paracasei* (LP) strain HF. A00232 5 × 10^9^ cfu/day for 8 weeks	IFN-γ, IL-4, IL-10, and TGF-β measured at baseline and weeks 8 and 12	-	-	→	Yes
**Atopic/allergic disorders: other**							
Taylor et al., 2006 Australia [34]	2-arm RCT; 118 newborns of women with a history of allergic disease and positive allergen skin prick test	Probiotic: *L. acidophilus* LAVRI-A1 3 × 10^9^ daily for first 6 months	IL-5, IL-6, TNF-α, IL-10, IL-13, and TGF- β measured at 6 months	Reduced IL-5 production in response to polyclonal stimulation (*p* = 0.044); reduced TNF-α responsiveness to house dust mite allergens (*p* = 0.046)	Reduced TGF-β production in response to polyclonal stimulation (*p* = 0.015); reduced IL-10 responses to tetanus toxoid vaccine antigen (*p* = 0.03) and house dust mite allergens (*p* = 0.014)	↑/↓	N/a
Chen et al., 2010 Taiwan [35]	2-arm RCT: 105 children aged 6–12 years with asthma and allergic rhinitis	Probiotic: *L. gasseri* PM-A0005 (A5) 2 × 10^9^ cells twice daily for 8 weeks	TNF-α, IFN-γ, IL-12p40, IL-10, and IL-13 produced by PBMCs stimulated with PHA, *Dermatophagoides pteronyssinus* (Der p), or Der p supplemented with *L. gasseri* A5 measured at baseline and 8 weeks	Decreased TNF-α in PBMCs in medium alone and stimulated with Der p. (*p* < 0.05). Decreased IFN-γ, IL-12 in PBMCs stimulated with PHA or Der p (*p* < 0.05).	Decreased IL-13 in medium alone and stimulated with PHA or Der p (*p* < 0.05)	↑/↓	Yes
Huang et al., 2018 Taiwan [36]	4-arm RCT: 160 children aged 6–18 years with asthma	Probiotic: *L. paracasei* GMNL-133 (BCRC 910520, CCTCC M2011331), *L. fermentum* GM-090 (BCRC 910259, CCTCC M204055), or both for 3 months (frequency and dose not specified)	IFN-γ, TNF α, and IL-4 measured at baseline and 3 months	→	→	→	Yes
**Autoimmune disorders: coeliac disease**							
Drabińska et al., 2019 Poland [24]	2-arm RCT: 34 children aged 4–17 years with coeliac disease	Prebiotic: 10 g of oligofructose-enriched inulin (Synergy 1) daily for three months	IL-1β, IL-1ra, IL-6, IL-8, IL-12p70, TNF-α, and IL-10 measured at baseline and 3 months	→	→	→	N/a
Olivares et al., 2014 Spain [37]	2-arm RCT: 33 children aged 2–17 years with newly diagnosed coeliac disease	Probiotic: *B. longum* CECT 7347 10⁹ cfu daily for 3 months	Lymphocyte subsets, TNF-α, IF-γ, IL-10, IL-13, and TGF-β measured at baseline and 3 months	Fall in CD3^+^ (*p* = 0.013) and HLA-DR^+^ T lymphocytes (*p* = 0.029) from baseline; decreased CD3^+^ T lymphocytes (*p* = 0.004) compared with controls	-	↓	Yes
Klemenak et al., 2015 Slovenia [38]	3-arm RCT: 49 children with coeliac disease compared to 18 healthy controls	Probiotic: *B. breve* BR03 and B632 2 × 10^9^ cfu daily for three months	TNF-α and IL-10 measured at baseline and 3 months	Decreased TNF-α levels from baseline (*p* < 0.05)	-	↓	N/a
**Autoimmune disorders: Other**							
Fortes et al., 2020 Brazil [39]	2-arm RCT: 4 children aged 2–17 years with compensated or partially compensated nephrotic syndrome and dyslipidaemia	Probiotic: *L. plantarum* Lp-G18 2.5 × 10^9^ cfu daily for 12 weeks	TNF-α and IL-10 measured at baseline and during and at the end of the study	-	-	→	No
Shukla et al., 2016 India [44]	2-arm RCT: 46 children aged 13–19 years with active enthesitis-related juvenile inflammatory arthritis	Probiotic: (*S. thermophilus*, *B. breve*, *B. longum*, *B. infantis*, *L. acidophilus*, *L. plantarum*, *L. paracasei* and *L. delbrueckii* (VSL#3) capsules 112·5 billion cells twice daily for 12 weeks	Blood Th1, Th2, Th17 and T_reg_ cells, ESR, CRP, IFN-γ, IL-6, IL-17, TNF-α, IL−4, and IL-10 measured at baseline and 12 weeks	Fall in IL-6 from baseline (*p* = 0·007)	Increased Th2 cells (*p* < 0.05) and IL-10 in placebo group (*p* = 0·013) from baseline	↓/→	No
**Preterm infants**							
Aly et al., 2017 Egypt [25]	4-arm RCT: 40 newborns with gestational age ≤ 34 weeks and age > 3 days	Prebiotic: different doses of medically graded bee honey daily for 14 days	CD4 and CD8 cells measured at baseline and 7 and 14 days	-	-	→	Yes
Fujii et al., 2006 Japan [40]	2-arm RCT: 19 preterm newborns	Probiotic: *B. breve* M-16V 10^9^ cells twice daily from several hours after birth until discharge	IL-4, IL-5, IL-6, IFN-γ, TGF-β1, and TNF-α measured at baseline and 14 and 28 days	Increased TGF-β1 from baseline on days 14 (*p* = 0.026) and 28 (*p* = 0.029); increased TGF-β1 on day 28 (*p* = 0.005)	-	↓	No
**Overweight/obesity**							
Nicolucci et al., 2017 Canada [26]	2-arm RCT: 42 overweight and obese children aged 7–12 years	Prebiotic: oligofructose-enriched inulin (Synergy1) 8 g/day for 16 weeks	CRP, IFNγ, IL-1β, IL-4, IL-6, IL-8, IL-10, IL-33, monocyte chemoattractant protein-1, TNF-α, and lipopolysaccharide measured at baseline and 16 weeks	Decreased IL-6 from baseline compared with controls (*p* = 0.01)	-	↓	Yes
Other diseases; severe illness							
Wang et al., 2018 China [41]	2-arm RCT: 80 children aged ≤14 years with acute lung injury	Probiotic: Eosinophil Lactobacillus 5 × 10^6^ cfu 3 times daily for 10 days	TNF-α and IL-6 measured at baseline and 10 days	Decreased TNF-α (*p* = 0.0005) and IL-6 (*p* = 0.0004)	-	↓	Yes
Angurana et al., 2018 India [45]	2-arm RCT: 100 children aged 3 months to 12 years with severe sepsis	Probiotic: VSL#3 (*L. paracasei* DSM 24734, *L. plantarum* DSM 24730, *L. acidophilus* DSM 24735, *L. delbrueckii* subsp. *bulgaricus* DSM 24734, *B. longum* DSM 24736, *B. infantis* DSM 24737, *B. breve* DSM 24732, *S. thermophilus* DSM 24731; Danisco-Dupont USA, Madison, WI); 450 billion bacteria twice daily for 7 days	IL-12p70, IL-6, IL-17, TNF-α, IL-10, and TGF -β1 measured on days 1 and 7	Decreased IL-6 (*p* = 0.001), IL-12p70 (*p* = 0.001); IL-17 (*p* = 0.01), TNF-α (*p* = 0.01] and increased IL-10 (*p* = 0.02), TGF-β1 (*p* = 0.01) in probiotic vs. controls at day 7. From baseline, fall in IL-6 (*p* = 0.001), IL-12p70 (*p*= 0.01], IL-17 (*p* = 0.01), TNF-α (*p* = 0.001); increase in IL-10 (*p* = 0.001) and TGF-β1 (*p* = 0.001)	-	↓	Yes
**Other diseases**							
de Freitas et al., 2018 Brazil [46]	3-arm RCT: children with cystic fibrosis received synbiotic (n = 22, mean (SD) age: 9.6 ± 2.8 years) vs. healthy controls (n = 17, mean age: 8.6 ± 3.0 years)	Synbiotic: *L. paracasei*, *L. rhamnosus*, *L. acidophilus*, *B. lactis* 10^8^–10^9^ cfu daily of each strain and FOS (5.5 g/day) (Lactofos^®^) for 90 days	High sensitivity CRP, IL-1β, IL-6, IL-8, IL-12, TNF-α, myeloperoxidase, nitric oxide metabolites (NOx), and IL-10 measured at baseline and 90 days	Fall from baseline in NOx (*p* = 0.030)	-	↓	No
Wang et al., 2015 China [43]	2-arm RCT: 60 children < 18 years with Hirschsprung’s disease at risk of enterocolitis	Probiotic: *Bifidobacterium*, *L. acidophilus*, and *E. faecalis*; (BIFICO; strains not specified) >10^8^ cfu daily for 4 weeks	T lymphocyte subsets, TNF-α, IFN-γ, IL-6, IL-10 unclear when measured	Increased CD4^+^ cells (*p* = 0.048) and CD4^+^/CD8^+^ ratio (*p* = 0.005) compared with controls; decreased TNF-α *p* < 0.01), IFN-γ (*p* = 0.029), IL-6 (*p* = 0.015); increased IL-10 (*p* = 0.011) compared with controls	-	↓	Yes
Ellis et al., 2013 USA [42]	2-arm RCT: 16 term infants with congenital heart disease at risk of necrotising enterocolitis	Probiotic: *B. longum* ssp. *infantis* ATCC 15,697 4.2 × 10^9^ cfu twice two daily for 8 weeks or until death or discharge if sooner	IFN-γ, IL-1β, IL-8, TNF-α, and IL-10 measured weekly	-	Increased IFN-γ (*p* = 0.007) and IL-1β (*p* = 0.04)	↑	N/a

^1^ ↑ = pro-inflammatory; ↓ = anti-inflammatory; ↑/↓ = both pro-and anti-inflammatory; → = no effect observed; ^2^ statisticially significant benefit in one or more primary or secondary clinical outcomes in the intervention group compared with controls; N/a = not applicable; no clinical outcomes reported, RCT—randomised controlled trial; GOS—galacto-oligosaccharides; PBMC—peripheral blood mononuclear cells; PHA—phytohemagglutinin.

## Data Availability

Data are contained within the article.
